# A dataset for preparing pristine graphene-palladium nanocomposites using swollen liquid crystal templates

**DOI:** 10.1038/sdata.2017.196

**Published:** 2017-12-19

**Authors:** Tripti Vats, Prem Felix Siril

**Affiliations:** 1 School of Basic Sciences, Indian Institute of Technology Mandi, Himachal Pradesh, India

**Keywords:** Nanoparticles, Synthesis of graphene

## Abstract

Pristine graphene (G) has not received much attention as a catalyst support, presumably due to its relative inertness as compared to reduced graphene oxide (RGO). In the present work, we used swollen liquid crystals (SLCs) as nano-reactors for graphene-palladium nanocomposites synthesis. The ‘soft’ confinement of SLCs directs the growth of palladium (Pd) nanoparticles over the G sheets. In this dataset we include all the parameters and details of different techniques used for the characterization of G, SLCs and synthesized G-Pd nanocomposites. The synthesized G-palladium nanocomposites (Pd-G) exhibited improved catalytic activity compared with Pd-RGO and Pd nanoparticles, in the hydrogenation of nitrophenols and C-C coupling reactions.

## Background & Summary

Sp^2^ bonded carbon atoms in a 2-D graphene sheet with less defects, best conductivity, high surface area and ability to adsorb molecules through π-π interaction offers a good candidature as support materials for different nanomaterials^1–3^. But its actual application for this purpose remains under explored due to the low reactivity of G. There have been some strategies for preparing nanocomposites of G. But most of these methods involved introduction of vacancies in graphene sheets by applying strain or through functionalization, which in turn degrades pristine properties of graphene^4,5^. It is evident that in each of these methods, a compromise has been made in the form of defect introduction in graphitic structure^6^. Interestingly, some theoretical and limited experimental studies pointed out that certain metal nanoparticles can form relatively strong bonding with G^7^. Hence, functionalization of G as such may not be essential to form stable nanocomposites having strong electro-mechanical coupling between the nanoparticles and the graphene sheets^8^. Keeping this in view, we have developed a method to make nanocomposites of Pd nanoparticles that are uniformly decorated on G using swollen liquid crystals (SLCs) as templates^9^.

SLCs are soft templates that are formed by the self-assembly of surfactants and they have already been used in the synthesis of various nanomaterials^9–11^. In our published research work, we trapped G in the infinitely long surfactant-stabilized oil tubes that are regularly arranged in brine solution along with a Pd-precursor to prepare the nanocomposites^9,11^. We found that the same method can be used to prepare nanocomposites of RGO also. In that work, we did thorough characterization of the synthesized nanocomposites along with G and SLC. Detailed characterization of each stage of nanocomposite fabrication was also done. Data from characterization techniques such as UV-Visible spectroscopy, transmission electron microscopy (TEM), polarised optical microscopy(POM), small angle X-ray scattering (SAXS), X-ray diffraction (XRD) were used. We have already explained the structure, morphology, and composition of the nanocomposites in the published work. However, we are presenting more detailed data obtained by POM, AFM and TEM analysis here especially on the different characterization conditions used during the analysis, a very important aspect of our experimental work.

## Methods

### Experimental design

The synthesis of G-Pd (GPd_0.001M_ and GPd_0.01M_) nanocomposites was achieved by using SLCs as nanoreactors^9^. The subscripted text refers to the precursor concentration in the oil phase of the mesophases. RGPd_0.001M_ and Pd nanoparticles (Pd_0.001M_) were also prepared using the same procedure.

### Sample preparation

#### Synthesis of pristine graphene

G was successfully prepared using liquid phase exfoliation method with the help of aqueous solution of a surfactant^12^. Some parameters such as surfactant concentration, sonication time and graphite concentration were optimized to get maximum product yield^13^. The surfactant solution was prepared by dissolving sodium dodecyl sulphate (SDS) in water and G was synthesized by sonicating graphite flakes in surfactant solution. It is important to know that dissolving surfactant at high concentration (>0.3 g ml^−1^) is difficult and the dissolution at such high concentrations can be achieved by heating. The temperature of oven was set at 60 °C prior to heating and sample was sealed in glass culture tubes to avoid atmospheric contamination and evaporation of water. Once, the surfactant is completely dissolved in water, measured quantities of graphite powder was added to it and ultrasonicated in a bath sonicator. The temperature of bath (water) during sonication should be maintained at room temperature (25 °C).

The resulting dispersion was left to stand for 24 h, letting the unstable and heavy aggregated flakes of G to settle down completely. The undisturbed solution was then centrifuged at 500 rpm for 90 min. The speed of centrifugation was carefully chosen to selectively deposit heavier flakes while avoiding the deposition of few-layer G. The top half of the solution was pipetted out and retained for the further use. The synthesis of RGO was done by using the modified Hummers method^11^.

#### Synthesis of the nanocomposites

The nanocomposites were synthesised in two steps: Graphene and metal salt were entrapped in the SLC followed by deposition of nanoparticles on graphene sheets by reacting with hydrazine vapour.

##### Preparation of SLCs

SLCs contain a regular network of parallel cylinders that are regularly spaced in a continuous aqueous salt solution. The diameters of these cylinders can be swollen with a nonpolar solvent. Hence, it is very important to control the concentration of each component and use them in proper ratios. Typically, the dispersion of G in SDS (4 ml, 0.1 mg ml^−1^) was used to prepare NaCl solution (0.1 M) which was further vortex mixed with Pd(dba)_2_ in toluene (6 ml, 1×10^−3^ M). The addition of co-surfactant (1-Pentanol) in the above solution plays a very important role in deciding the stability and phase transition of hexagonal pattern of SLCs. The introduction of a co-surfactant should be done in small aliquots of 10 μl. The sample should be strongly vortex mixed followed by centrifugation. SLCs containing RGO and Pd(dba)_2_ were synthesized by following the similar procedure.

##### Formation of nanocomposites

Growth of nanoparticles was induced by exposing the mesophases to hydrazine vapour. The mesophases were transferred into small glass vials (10 ml) and the vials were kept in slightly bigger jars containing small amount of hydrazine. The bigger jars should be sealed tightly with glass cap and paraffin film to create the hydrazine vapour environment. It is important to mention that hydrazine is very toxic and hence needs proper precaution while handling. Proper clothes, laboratory aprons, gloves and mask should be worn while handling hydrazine. The reactions must be carried inside fume hood to avoid exposure. The progress of the reaction can be monitored by visible change in colour of the mesophases from transparent maroon coloured viscous gel to black gel.

The prepared nanocomposites or nanoparticles were collected by extraction using iso-propyl alcohol (IPA). The samples were washed copiously using IPA/water mixture (50:50) for the removal of surfactant from the nanoparticles.

### Analysis techniques

Atomic force microscopy (AFM): AFM analyses of G samples and GPd_0.001M_ were performed in non-contact mode on a commercial Agilent SPM 5,500 with suitable probes in a temperature-controlled room (25 °C) under atmospheric conditions. The data produced during this study have been deposited at figshare and are mentioned in Data Citations section (Data Citation 1). Samples were prepared by drop casting aqueous dispersion of G over silicon wafers. It is very important to completely dry the sample using dryer before doing the analysis. It is preferable to use fresh silicon wafer each time to reduce the effect of impurity while doing the analysis. AFM scans of similar areas were acquired at a maximum resolution of 512×512 pixels and at a rate of 0.5–1 Hz. The scanned areas were perfect squares that ranged in size from 2.5×2.5 μm^2^ (lower magnification) for G samples. We used 5,500 utilizes Keysight’s PicoView software for imaging and analysis of samples. This software allows a complete control over all scanning parameters and provides the flexibility required for more complex experiments. It provides exceptional ease of use and adaptability for critical-to-quality measurements in high-volume measurement applications non-contact AFM height measurements of graphene are not fully reliable due to the different mechanical properties of the graphene and the substrate. Depending on imaging parameters, the apparent height can substantially change.

AFM Tip specification: Tip was made of Antimony (n) doped Silicon having resistivity of 0.01–0.025 Ohm-cm. Shape of the tip was triangular while thickness was 4 μm, length was 125 μm and mean width was 40 μm. It normally operates on 320 kHz resonance frequency and Force constant was 42 N/m. The tip has no coating on the front side while back side was covered with a thin layer (50+/−10 nm) AI.

POM (Polarized optical microscopy): The synthesized mesophases were first characterized by polarized optical microscopy (POM) imaging and results are uploaded in data repository (figshare), images 24hr_1_POM, 24hr_2_POM, 24hr_3_POM and 24hr_4_POM were the optical images of SLC taken after 24 h. The link to data repository is addressed in data citation section (Data Citation 2). The focal conic texture is the important characteristic of the hexagonal liquid crystals. The samples were prepared by depositing small amount of SLC directly taken out from the sealed tube on a glass slide and was sandwiched with a cover slip. Small amount of vacuum grease was applied to the edges of the coverslip to prevent the evaporation of the solvents and to protect the mesophases from collapsing. POM imaging of the SLCs was carried out using Nikon ECLIPSE LV 100 POL microscope. A high intensity 50 W halogen light source was used for optical microscopy. The lens was incorporated as fly-eye lens design that gives output more than a 100 W lamp while reducing the power consumption and heat generation, thereby reducing the chance of heat-induced focus drift. The stage with mounted sample was pre-adjusted parallel to lens. CFI P Achromat 20 X objective lens was used for imaging. Nikon imaging software NIS-ElEMENTS BR was used for capturing and processing of images. Digital colour cooled camera DS-Ri1 operating at 100 V was used to capture the images with 12.7 million pixels resolution (2,200 TV lines).

Transmission electron Microscopy (TEM): Samples were prepared for TEM analysis by dropping aqueous suspension of G and its nanocomposites using a micropipette onto the surface of a copper TEM grid coated with an amorphous carbon film. Once the solution had dried, imaging was done using Tecnai 200 kV D2224 Super Twin microscope using LaB6 gun operating at an acceleration voltage of 200 kV. Selected TEM images are available at figshare (Data Citation 3), and the data acquisition conditions can be found in the ISA-Tab metadata record for this Data Descriptor.

## Data Records

All data records listed in this Data Descriptor are available from the figshare repository. The results obtained through AFM analysis of synthesized G sheets are deposited under the caption name AFM images (Data Citation 1). The POM images of SLC are deposited as Polarized Optical Microscopy (POM) images (Data Citation 2). The TEM images of synthesized G and nanocomposites are deposited in the folder named as Transmission electron microscopy (TEM) images (Data Citation 3).

## Technical Validation

The experimental design presented in this dataset has been validated in several distinct ways. AFM analysis of graphene samples and nanocomposite were done by using same tip geometry and cantilever spring constant. The sample depositions were done on the silicon wafers taken from the set of pack. Moisture adds disability to the SLC so sealing the sample holder while performing different characterization is mandatory. The electron microscopes used to acquire the datasets described in this paper were professionally maintained and aligned for optimal imaging conditions prior to dataset acquisition.

## Additional information

**How to cite this article:** Vats, T. & Siril, P. F. A dataset for preparing pristine graphene-palladium nanocomposites using swollen liquid crystal templates. *Sci. Data* 4:170196 doi: 10.1038/sdata.2017.196 (2017).

**Publisher’s note:** Springer Nature remains neutral with regard to jurisdictional claims in published maps and institutional affiliations.

## Supplementary Material


